# Risk scores to predict mortality 2 and 5 years after surgery for colorectal cancer in elderly patients

**DOI:** 10.1186/s12957-021-02356-6

**Published:** 2021-08-26

**Authors:** Nerea González, Ane Loroño, Urko Aguirre, Santiago Lázaro, Marisa Baré, Maximino Redondo, Eduardo Briones, Cristina Sarasqueta, Amaia Bilbao, Nerea Fernández de Larrea, José María Quintana, for the REDISSEC-CARESS/CCR group, Jose María Quintana, Marisa Baré, Maximino Redondo, Eduardo Briones, Nerea Fernández de Larrea, Cristina Sarasqueta, Antonio Escobar, Francisco Rivas, Maria M. Morales-Suárez-Varela, Juan Antonio Blasco, Isabel del Cura, Inmaculada Arostegui, Irantzu Barrio, Amaia Bilbao, Nerea González, Susana García-Gutiérrez, Iratxe Lafuente, Urko Aguirre, Miren Orive, Josune Martin, Ane Antón-Ladislao, Núria Torà, Marina Pont, María Purificación Martínez del Prado, Alberto Loizate, Ignacio Zabalza, José Errasti, Antonio Z. Gimeno, Santiago Lázaro, Mercè Comas, Jose María Enríquez, Carlos Placer, Amaia Perales, Iñaki Urkidi, Jose María Erro, Enrique Cormenzana, Adelaida Lacasta, Pep Piera, Elena Campano, Ana Isabel Sotelo, Segundo Gómez-Abril, F. Medina-Cano, Julia Alcaide, Arturo Del Rey-Moreno, Manuel Jesús Alcántara, Rafael Campo, Alex Casalots, Carles Pericay, Maria José Gil, Miquel Pera, Pablo Collera, Josep Alfons Espinàs, Mercedes Martínez, Mireia Espallargues, Caridad Almazán, Paula Dujovne Lindenbaum, José María Fernández-Cebrián, Rocío Anula, Julio Mayol, Ramón Cantero, Héctor Guadalajara, María Alexandra Garceau, Damián García, Mariel Morey, Alberto Colina

**Affiliations:** 1 Osakidetza Basque Health Service, Galdakao – Usansolo Hospital (Research Unit), Galdakao, Basque Country Spain; 2 Kronikgune Institute for Health Services Research, Barakaldo, Basque Country Spain; 3 Health Services Research on Chronic Patients Network, REDISSEC, Galdakao, Basque Country Spain; 4 Osakidetza Basque Health Service, Galdakao–Usansolo Hospital (Surgery Department), Galdakao, Basque Country Spain; 5 Clinical Epidemiology and Cancer Screening, Parc Taulí University Hospital, Parc del Taulí, 1, 08208 Sabadell, Barcelona, Spain; 6 Andalusian Health Service, Resarch Unit, Costa del Sol Hospital, Autovía A-7 Km, 187-29603 Marbella, Malaga Spain; 7 UDG Public Health, AP Sevilla district, Av. de Jerez, 41013 Sevilla, Spain; 8 Biodonostia Health Research Institute, Donostia Universitary Hospital, Begiristain Doktorea Pasealekua, 20014 Donostia-San Sebastian, Guipuzkoa Spain; 9 Osakidetza Basque Health Service, Research Unit, Basurto Universitary Hospital, Montevideo Etorb., 18, 48013 Bilbao, Bizkaia Spain; 10 Epidemiology National Centre, Institute of Health Carlos III, Calle de Melchor Fernández Almagro, 5, 28029 Madrid, Spain; 11 CIBER of Epidemiology and Public Health (CIBERESP), Madrid, Spain

**Keywords:** Colorectal cancer, Elderly, Surgery, Outcome, Mortality, Risk score

## Abstract

**Background:**

The aim of this study was to identify predictors of mortality in elderly patients undergoing colorectal cancer surgery and to develop a risk score.

**Methods:**

This was an observational prospective cohort study. Individuals over 80 years diagnosed with colorectal cancer and treated surgically were recruited in 18 hospitals in the Spanish National Health Service, between June 2010 and December 2012, and were followed up 1, 2, 3, and 5 years after surgery. Sociodemographic and clinical data were collected. The primary outcomes were mortality at 2 and between 2 and 5 years after the index admission.

**Results:**

The predictors of mortality 2 years after surgery were haemoglobin ≤ 10 g/dl and colon locations (HR 1.02; CI 0.51–2.02), ASA class of IV (HR 3.55; CI 1.91–6.58), residual tumour classification of R2 (HR 7.82; CI 3.11–19.62), TNM stage of III (HR 2.14; CI 1.23–3.72) or IV (HR 3.21; CI 1.47–7), LODDS of more than − 0.53 (HR 3.08; CI 1.62–5.86)) and complications during admission (HR 1.73; CI 1.07–2.80). Between 2 and 5 years of follow-up, the predictors were no tests performed within the first year of follow-up (HR 2.58; CI 1.21–5.46), any complication due to the treatment within the 2 years of follow-up (HR 2.47; CI 1.27–4.81), being between 85 and 89 and not having radiotherapy within the second year of follow-up (HR 1.60; CI 1.01–2.55), no colostomy closure within the 2 years of follow-up (HR 4.93; CI 1.48–16.41), medical complications (HR 1.61; CI 1.06–2.44), tumour recurrence within the 2 years of follow-up period (HR 3.19; CI 1.96–5.18), and readmissions at 1 or 2 years of follow-up after surgery (HR 1.44; CI 0.86–2.41).

**Conclusion:**

We have identified variables that, in our sample, predict mortality 2 and between 2 and 5 years after surgery for colorectal cancer older patients. We have also created risks scores, which could support the decision-making process.

**Trial registration:**

ClinicalTrials.gov, NCT02488161.

## Introduction

Populations are ageing all around the world. In Spain, in 2016, 18% of the population was over 60 years, with octogenarians representing 6% of the total, and these percentages will grow in the coming years [1].

Cancer is one of the main causes of morbidity and mortality worldwide, with 18 million new cases and 9.6 million deaths in 2018 [2]. Colorectal cancer was the most frequent cancer, with 1.8 million new cases and almost 861,000 deaths [2]. As for the incidence of cancer in the elderly, a review stated that it is 11-fold compared with younger patients [3]. There is therefore an increase in the average age at the time of cancer diagnosis.

The relationship between age and mortality due to cancer is complex, as it can be confounded by other factors, such as differences in stage at presentation, tumour site, or type of treatment received [4, 5]. Therefore, it becomes important to have systems of stratification of these patients to see who could have a better long-term prognosis and/or who could benefit from certain treatments.

Several publications have described factors influencing mortality in the older patients due to colorectal cancer [6–8], but most of this research has focused on the short term. Studies that have analysed factors in the long term have identified complications, American Society of Anaesthesiologists (ASA) class, tumour stage, or increased age as having a significant influence on mortality in older patients [5, 9–13]. Nevertheless, the majority of these studies have employed multivariable analysis, without any classification or scoring system. The aim of this study was to detect variables that have the most weight in the prediction of mortality in older colorectal cancer patients and to develop a risk score to stratify this population.

## Materials & methods

The data presented in this manuscript is a post hoc analysis that comes from a prospective observational cohort study that recruited patients diagnosed with colorectal cancer who were treated surgically. Patients were recruited in 18 hospitals in the Spanish National Health Service, between June 2010 and December 2012, and they were followed up 1, 2, and 5 years after the surgery.

The inclusion criteria were that patients were diagnosed with cancer of the colon or rectum (between the anal margin and 15 cm above it), had curative or palliative surgery performed for first time, and signed the informed consent form to participate in the study. For this manuscript, we added an age criterion, selecting patients 80 years or older. Patients were excluded if they had in situ cancer, inoperable tumours, a severe mental or physical condition that prevented the patient from responding to questionnaires, or terminal illness.

Patients were identified from the surgical waiting lists and were invited to participate during a clinical appointment or by letter. After this selection process, clinical data were collected at baseline, 1 month, and 1, 2, and 5 years after the surgery.

Patients were informed of the objectives of the study, and they were asked to provide written informed consent before inclusion. The Institutional Review Boards of the participating hospitals approved this project. More details of this study can be found in an earlier publication [14].

### Data collection

Data were collected from the medical records by trained reviewers, employing data collection forms and an instruction manual to ensure consistency.

Patients who did not survive within the 30-day period after the index surgery were excluded in this study.

*Baseline data* included sociodemographic characteristics; clinical history; preoperative findings, in particular, laboratory test results, diagnostic test results, and tumour site; and data from the outpatient preoperative anaesthesia appointment, including ASA class [15].

*Data related to hospital admission* included data on the surgical intervention; histopathological data, including TNM stage; residual tumour classification after surgery; number of organ invasion; lymph node involvement, expressed as the log odds of positive lymph nodes (LODDS) [16]; length of stay; presence and degree of complications, grouped in surgical, medical, infectious, and haematological (haemorrhage/thrombosis/embolism); types of treatment given, including need for reintervention; and death.

Finally, data were collected on relevant variables *up to 30 days after surgery* (laboratory and diagnostic test results, presence of complications, readmissions, reintervention, or death) *and through the first, second, and fifth postoperative years* (radiation therapy, chemotherapy, laboratory and diagnostic test results, colostomy closure within the 2 years follow-up, presence of complications, tumour recurrence, readmission or reoperation, and death).

### Outcome measures

The primary outcomes were mortality at 2 and between 2 and 5 years after the patient was first admitted to the hospital for surgery for colorectal cancer (index admission). Vital status was established by reviewing medical records and examining the hospital database and public registers of deaths. Deaths were considered confirmed if the name, sex, date of birth, and identity card number on the record matched those of the participant.

### Statistical analysis

Descriptive statistics of the retrieved variables were calculated using means and standard deviations (SD) and median and interquartile ranges for quantitative data and frequencies and percentages for categorical variables. Chi-square and Fisher’s exact tests were used for comparing categorical variables and Student’s *t* test or the nonparametric Wilcoxon test for assessing the relationship of mortality up to 2 and between 2 and 5 years with potentially relevant continuous variables.

In the multivariable analysis, a Cox regression model was developed that used mortality up to 2 years and between 2 and 5 years (excluding those patients who did not survive at 2 years) as the dependent variable. In addition, the hospital effect was added to the model, in order to analyse any variation caused by differences between centres. When that effect showed no statistical significant differences, it was removed from the model. The goodness of fit of the models was assessed with the Greenwood-Nam-D'Agostino (GND) test [17] and the C-index.

Two mortality risk scores were developed, one for each of the studied outcomes. To develop these predictive risk scores and therefore, to determine the scoring weights related to the variable categories of each predictor involved in the punctuation, we first assigned a weight to each risk factor in relation to each β parameter based on the multivariate Cox regression model; as the first step, regression coefficients that turned out to be statistically significant were selected. The smallest beta coefficient was identified to divide each of the significant betas by this value. The resulting value led to the corresponding weight of each of the predictor categories. Then, weights of each of the risk factors presented by a patient were summed, with a higher score corresponding to a higher likelihood of death. Considering the optimal cut-points [18], three severity categories were created for each score, and Kaplan–Meier curves were plotted for each risk group. As for internal validations of our models, a total of 500 bootstrap samples were generated to calculate the C-index and their corresponding confidence intervals of the scores. The GND test was also used to assess the goodness of fit of the models.

All effects were deemed statistically significant at *p* < 0.05. All statistical analyses were performed using SAS Software, version 9.4 (SAS Institute, Inc., Carey, NC, USA), and figures were depicted using R statistical software, version 3.5.

## Results

The sample of patients aged 80 years or older was comprised of 426 individuals, of whom, 21 (4.92%) died within the first 30 days after the index surgery. Out of those who fulfilled the criteria (*n* = 405), 92 (22.71%) died during the first 2 years and a further 99 (31.63%) between the second and the fifth year of follow-up. These 405 patients had a mean age of 83.24 years (SD 2.93) and the 92% had an ASA classification lower than class IV. Descriptive data and univariate analyses are shown in Table 1.

**Table 1:** Descriptive and univariable analyses

	Descriptive analysis	Unadjusted analysis			
		Mortality up to 2 years		Mortality within 2–5 years	
	*n* (%)	HR (95% CI)	***p*** value	HR (95% CI)	***p*** value
Age (years)^*^	83.24 (2.93)	1.08 (1.02, 1.15)	0.01	1.04 (0.97, 1.11)	0.28
BMI^*^	27.20 (4.04)	1 (0.94, 1.06)	0.99	0.98 (0.92, 1.03)	0.40
**BMI**					
≤ 25	101 (23.71)	Reference		Reference	
25–30	156 (36.62)	0.96 (0.56, 1.67)	0.89	0.97 (0.59, 1.58)	0.90
> 30	72 (16.90)	0.87 (0.44, 1.74)	0.70	0.69 (0.36, 1.32)	0.26
**Smoking status**					
Non-smoker	197 (55.49)	Reference			
Current smoker	14 (3.94)	0.67 (0.16, 2.75)	0.57	0.48 (0.12, 1.97)	0.31
Ex-smoker	144 (40.56)	1.38 (0.91, 2.10)	0.13	1.20 (0.79, 1.81)	0.39
**Alcoholism**					
No	315 (91.30)	Reference		Reference	
Yes	30 (8.70)	1.86 (0.99, 3.49)	0.06	1.19 (0.55, 2.58)	0.65
**Charlson comorbidity index**					
≤ 5	389 (91.31)	Reference		Reference	
> 5	37 (8.69)	2.03 (1.13, 3.64)	0.02	2.36 (1.26, 4.42)	0.008
**Haemoglobin at baseline (g/dl) and localisation**					
> 10 and rectum cancer	93 (21.93)	1.18 (0.69, 2.02)	0.54	0.99 (0.60, 1.65)	0.98
> 10 and colon cancer	250 (58.96)	Reference		Reference	
≤ 10 and rectum cancer	7 (1.65)	1.69 (0.41, 6.96)	0.47	0.68 (0.09, 4.9)	0.70
≤ 10 and colon cancer	74 (17.45)	2.36 (1.46, 3.83)	0.0001	1.44 (0.84, 2.48)	0.19
Length of stay, days^**^	11 (8-17)	1.03 (1.02, 1.04)	< 0.001	1.02 (1.01, 1.04)	0.010
**ASA**					
I, II, III	380 (91.79)	Reference		Reference	
IV	34 (8.21)	2.28 (1.27, 4.11)	0.006	1.24 (0.57, 2.67)	0.58
**Invasion: vascular, perineural, lymphatic**					
0, 1	374 (87.79)	Reference		Reference	
2, 3	52 (12.21)	2.45 (1.25, 4.78)	< 0.001	1.88 (1.03, 3.44)	0.04
**Residual tumour classification**					
R0	376 (91.48)	Reference		Reference	
R1	19 (4.62)	2.21 (0.96, 5.10)	0.06	3.87 (1.79, 8.37)	< 0.001
R2	16 (3.89)	11.15 (6.09, 20.40)	< 0.001	19.72 (4.63, 84.08)	< 0.001
**TNM**					
0, I, II	248 (58.77)	Reference		Reference	
III	140 (33.18)	2.24 (1.39, 3.59)	< 0.001	1.30 (0.85, 1.99)	0.23
IV	34 (8.06)	6.55 (3.72, 11.53)	< 0.001	1.73 (0.70, 4.32)	0.24
**LODDS**					
Less than − 1.36	329 (82.66)	Reference		Reference	
(− 1.36, − 0.53]	34 (8.54)	2.96 (1.60, 5.43)	< 0.001	2.49 (1.32, 4.68)	0.005
Greater than − 0.53	35 (8.79)	4.73 (2.73, 8.21)	< 0.001	1.90 (0.86, 4.21)	0.11
**Recurrence of the tumour up to 1 year**					
No	357 (87.71)	Reference		Reference	
Yes	50 (12.29)	5.07 (3.28, 7.83)	< 0.001	2.31 (1.17, 4.60)	0.02
**Complications during admission**					
No	187 (43.90)	Reference		Reference	
Yes	239 (56.10)	1.48 (0.97, 2.25)	0.07	1.22 (0.82, 1.81)	0.34
**Readmission within 30 days**					
No	382 (91.39)	Reference		Reference	
Yes	36 (8.61)	1.77 (0.97, 3.25)	0.06	1.87 (0.99, 3.50)	0.051
**Pre-intervention radiotherapy**					
No	390 (91.55)	Reference		Reference	
Yes	36 (8.45)	0.44 (0.16, 1.21)	0.11	0.55 (0.24, 1.26)	0.16
**Post-intervention radiotherapy**					
No	416 (97.65)	Reference		Reference	
Yes	10 (2.35)	1.32 (0.42, 4.15)	0.64	0.43 (0.06, 3.11)	0.41
**Pre-intervention chemotherapy**					
No	380 (93.83)	Reference		Reference	
Yes	25 (6.17)	0.32 (0.08, 1.30)	0.11	0.49 (0.18, 1.31)	0.15
**Post-intervention chemotherapy**					
No	345 (58.19)	Reference		Reference	
Yes	60 (14.81)	1.31 (0.76, 2.25)	0.33	1.06 (0.60, 1.87)	0.84
**Age at baseline & radiotherapy treatment within the 2-year follow-up**					
< 85 years	300 (70.42)	Reference		Reference	
85–89 years with no radiotherapy	100 (23.47)	1.61 (1.03, 2.53)	0.04	1.69 (1.08, 2.63)	0.02
85–89 years with radiotherapy or ≥ 90 years	26 (6.10)	1.28 (0.55, 2.96)	0.57	0.81 (0.29, 2.22)	0.68
**Diagnosis tests performed within the first year of follow-up**					
No	104 (24.41)	Reference		Reference	
Yes	322 (75.59)	0.71 (0.44, 1.14)	0.15	0.62 (0.39, 0.98)	0.04
**Any complication due to the treatment within the 2-year follow-up**					
No	390 (91.55)	Reference		Reference	
Yes	36 (8.45)	2.15 (1.21, 3.80)	0.009	0.46 (0.24, 0.86)	0.01
**Colostomy closure within the 2-year follow-up period**					
No	401 (94.13)	1.56 (0.57, 4.26)	0.38	2.57 (0.81, 8.10)	0.11
Yes	25 (5.87)	Reference		Reference	
**Medical complications within the 2-year follow-up period**					
No	219 (51.41)	Reference		Reference	
Yes	207 (48.59)	1.47 (0.97, 2.21)	0.07	1.84 (1.23, 2.75)	0.003
**Tumour recurrence within the 2-year follow-up period**					
No recurrence	303 (82.11)	Reference		Reference	
Yes	66 (17.89)	5.50 (3.22, 9.39)	< 0.001	3.16 (1.97, 5.04)	< 0.001
**Readmissions within the 2-year follow-up period**					
No	284 (69.78)	Reference		Reference	
Only at 1 year or at 2nd year after the surgical intervention	105 (25.80)	3.22 (2.09, 4.95)	< 0.001	1.43 (0.89, 2.29)	0.14
At both measurements	18 (4.42)	4.14 (1.94, 8.83)	< 0.001	2.24 (0.90, 5.54)	0.08

*HR* hazard ratio, *CI* confidence interval. ^*^Mean (SD)**.** ^**^Median (p25, p75). *ASA* American Society of Anaesthesiologists Physical Status Classification System, *TNM* cancer stage, *LODDS* log odds of positive lymph nodes

The independent predictors of mortality in patients 80 years or older 2 years after surgery were having haemoglobin ≤ 10 g/dl and colon cancer (vs haemoglobin > 10 and colon cancer), an ASA class of IV (vs I, II, III), a residual tumour classification of R2 (vs R0), a TNM stage of III or IV (vs 0, I, II), LODDS of more than – 0.53 (vs less than − 1.36), and complications during admission (Table 2). The model showed good discrimination, with a C-index of 0.80 (95% CI: 0.78–0.87). Likewise, the GND test was < 0.001.

**Table 2:** Multivariable analysis of mortality at 2 years

Effects	Beta (s.e.)	HR (95% CI)	*p* value	Scoring weight
Hgb (g/dl) at baseline and localisation				
Hgb > 10 and rectum (*n* = 67)	0.02 (0.35)	1.02 (0.51, 2.02)	0.96	0
Hgb > 10 and colon (*n* = 231)	Reference			0
Hgb ≤ 10 and rectum (*n* = 6)	− 0.31 (0.78)	0.74 (0.16, 3.36)	0.69	0
Hgb ≤ 10 and colon (*n* = 62)	0.90 (0.26)	2.45 (1.47, 4.10)	< 0.001	4
ASA class				
I, II, III (*n* = 334)	Reference			0
IV (*n* = 32)	1.27 (0.32)	3.55 (1.91, 6.58)	< 0.001	5
Residual tumour classification				
R0 (*n* = 341)	Reference			0
R1 (*n* = 16)	0.30 (0.46)	1.34 (0.55, 3.28)	0.52	0
R2 (*n* = 9)	2.06 (0.47)	7.82 (3.11, 19.62)	< 0.001	7
TNM				
0, I, II (*n* = 218)	Reference			0
III (*n* = 120)	0.76 (0.28)	2.14 (1.23, 3.72)	0.007	3
IV (*n* = 28)	1.17 (0.40)	3.21 (1.47, 7)	0.003	4
LODDS				
Less than or equal to − 1.36 (*n* = 309)	Reference			0
(− 1.36, − 0.53] (*n* = 30)	0.55 (0.35)	1.73 (0.87, 3.43)	0.12	0
Greater than − 0.53 (*n* = 27)	1.12 (0.33)	3.08 (1.62, 5.86)	< 0.001	4
Complications during admission				
No (*n* = 163)	Reference			0
Yes (*n* = 203)	0.55 (0.25)	1.73 (1.07, 2.80)	0.03	2
C-index^*^	0.80 (0.78, 0.87)			
GND test^*^	< 0.001			

*Hgb* haemoglobin (g/dl), *Beta (s.e.)* regression coefficient and its standard error, *HR* hazard ratio, *CI* confidence interval, *C-index* concordance index, *GND* the Greenwood-Nam-D'Agostino test, *Reference*: reference category. ^*^Hospital-adjusted values

Between the second and the fifth year of follow-up, the predictors of mortality were no tests performed within the first year of follow-up (vs CAT, colonoscopy and CAT + colonoscopy), any complication due to the treatment within the 2 years of follow-up, being between 85 and 89 and not having radiotherapy within the second year of follow-up, not having the colostomy closed within the 2 years of follow-up, having medical complications, tumour recurrence within the 2 years of follow-up period, and having readmissions at 1 or 2 years of follow-up (Table 3). In this model, the C-index was 0.73 (95% CI 0.68–0.78), which demonstrates good discrimination of the model. The GND test was < 0.001.

**Table 3:** Multivariable analysis of mortality at 5 years

Effects	Beta (s.e.)	HR (95% CI)	***p*** value	Scoring weight
**Diagnosis tests performed within the first year of follow-up**				
No (*n* = 96)	0.95 (0.38)	2.58 (1.21, 5.46)	0.01	4
CAT (*n* = 152)	0.48 (0.37)	1.62 (0.79, 3.32)	0.19	0
Colonoscopy (*n* = 15)	0.49 (0.61)	1.62 (0.50, 5.33)	0.42	0
CAT + Colonoscopy (*n* = 50)		Reference		0
**Any complication due to the treatment within the 2-year follow-up**				
No (*n* = 292)	Reference			0
Yes (*n* = 21)	0.90 (0.34)	2.47 (1.27, 4.81)	0.008	4
**Age at baseline & radiotherapy treatment within the 2-year follow-up**				
< 85 years (*n* = 230)	Reference			0
85–89 year with no radiotherapy (*n* = 65)	0.47 (0.24)	1.60 (1.01, 2.55)	0.047	2
85–89 year with radiotherapy or ≥ 90 years (*n* = 18)	− 0.33 (0.52)	0.72 (0.26, 1.99)	0.53	0
**Colostomy closure within the 2-year follow-up period**				
No (*n* = 292)	1.60 (0.61)	4.93 (1.48, 16.41)	0.01	9
Yes (*n* = 21)	Reference			0
**Medical complications within the 2-year follow-up period**				
No (*n* = 173)	Reference			0
Yes (*n* = 140)	0.48 (0.21)	1.61 (1.06, 2.44)	0.03	2
**Tumour recurrence within the 2-year follow-up period**				
No recurrence (*n* = 274)	Reference			0
Yes (*n* = 39)	1.16 (0.25)	3.19 (1.96, 5.18)	< 0.001	5
**Readmissions within the 2-year follow-up period**				
No (*n* = 240)	Reference			0
Only at 1 year or at 2nd year after the surgical intervention (*n* = 63)	0.37 (0.26)	1.44 (0.86, 2.41)	0.16	0
At both measurements (*n* = 10)	1.21 (0.50)	3.35 (1.27, 8.85)	0.02	5
C-index^*^	0.73 (0.68, 0.78)			
GND test^*^		< 0.001		

*Beta (s.e.)* regression coefficient and its standard error, *HR* hazard ratio, *CI* confidence interval, *C-index* concordance index, *GND* the Greenwood-Nam-D'Agostino test, *Reference* reference category. ^*^Hospital-adjusted values

Table 4 shows that the risk of 2 years mortality was significantly higher in those patients whose score was ≥ 8 (hazard ratio, 10.50; 95% CI, 6.02–18.29; *p* < 0.001) and for patients with a score of 4–7 (hazard ratio, 3.08; 95% CI, 1.71–5.55; *p* < 0.001), compared to those in the lowest risk group. With regards to the 2- to 5-year mortality risk, it was higher in the patients with a score ≥ 17 (hazard ratio, 6.10; 95% CI, 3.19–11.66; *p* < 0.001) and in the moderate risk group (hazard ratio, 1.99; 95% CI, 1.15–3.45; *p* = 0.015), compared with the patients with the lowest risk.

**Table 4:** Risk score stratifications of mortality at 2 and 5 years

	No. event/no. at risk	HR (95% CI)	*p* value
Mortality at 2 years			
Risk score (continuous)	-	1.33 (1.26, 1.41)	< 0.001
Risk score (categorical)			
0–3 (low)	20/213 (9.39)	Reference	
4–7 (moderate)	25/99 (25.25)	3.08 (1.71, 5.55)	< 0.001
≥ 8 (high)	34/54 (62.96)	10.50 (6.02, 18.29)	< 0.001
C-index^*^	0.80 (0.76, 0.85)		
GND test^*^	< 0.001		
Mortality at 5 years			
Risk score (continuous)	-	1.27 (1.20, 1.36)	< 0.001
Risk score (categorical)			
0–10 (low)	16/92 (17.39)	Reference	
11–16 (moderate)	61/190 (32.11)	1.99 (1.15, 3.45)	0.015
≥ 17 (high)	22/31 (70.97)	6.10 (3.19, 1)	< 0.001
C-index^*^	0.72 (0.67, 0.77)		
GND test	< 0.001		

There are pairwise-statistical significant differences among the three established categories in both developed risk scores. *Reference* reference category, *No. event/no.at risk* number of events/no. of patients in the risk level, *HR* hazard ratio, *CI* confidence interval, *C-index* concordance index, *GND* the Greenwood-Nam-D'Agostino test

^*^Hospital-adjusted values

**Fig. 1:**
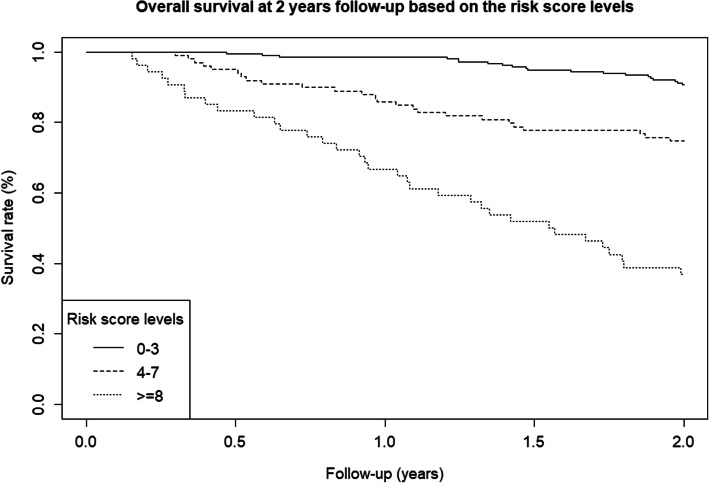
Kaplan–Meier survival curve 2 years after surgery for the three mortality risk groups

**Fig. 2:**
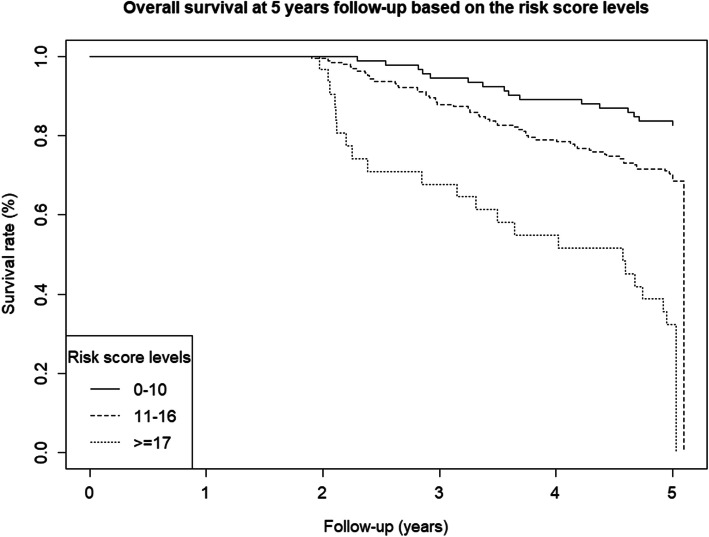
Kaplan–Meier survival curve between 2 and 5 years after surgery for the three mortality risk groups

This same pattern can be seen in the Kaplan–Meier curves, shown in the Online Resource. The probability of surviving 2 years after the intervention was below 0.4 for patients classified as having a high risk of death, rising to near 0.8 for those with moderate risk and higher for patients with a low risk of mortality. The probabilities of surviving between the second and the fifth year of follow-up years after the surgery were 0.4, 0.8, and 0.9 for each risk group, respectively (Figs. 1 and 2).

Internal validation of all the models were performed where it can be seen that those variables selected for our two models are the ones which were selected more frequently by bootstrap (Table 5).

**Table 5:** Results of the bootstrap analysis for the variable selection in the models

	Mortality to 2 years (%)	Mortality to 5 years (%)
Hgb (g/dl) at baseline and localisation	26.56	-
ASA class	9.90	-
**Residual tumour classification**	19.78	-
TNM	16.32	-
LODDS	17.76	3.19
Complications during admission	6.27	-
Invasion: vascular, perineural, lymphatic	3.41	1.82
Diagnosis tests performed within the first year of follow-up	-	22.61
Any complication due to the treatment within the 2 year follow-up	-	9.46
Age at baseline & Radiotherapy treatment within the 2 year follow-up	-	12.60
**Colostomy closure within the 2-year follow-up period**	-	9.65
Medical complications within the 2-year follow-up period	-	10.27
Tumour recurrence within the 2-year follow-up period	-	14.50
Readmissions within the 2-year follow-up period	-	15.89

% percentage, - not applicable

## Discussion

This study has identified variables that, in our sample, predict mortality at 2 and between 2 and 5 years after surgery for colorectal cancer, in patients 80 years or older. In addition, we have created risk scores for each follow-up time point, which can help classify patients by mortality risk.

Some of these variables have also been found to be mortality predictors in other studies, namely, cancer stage [9–11, 13], ASA class [7, 9–11], LODDS [19], tumour recurrence [20], and complications [9, 10, 21], with similar results to those found in our study.

Regarding haemoglobin, Kim and Kim [11] did not find it to be predictive of mortality in their sample, and in other studies, it was evaluated as predictor of mortality and/or complications on the short term [22, 23].

We have not found studies analysing two other variables identified in our models, i.e., residual tumour classification and colostomy closure. The residual tumour classification is usually employed to assess the presence of tumour tissue remaining after surgery [21]. As has been found in our study, patients with a poorer response to treatment, reflected in a higher score in residual tumour classification, generally have a poorer prognosis [21]. As far as colostomy closure is concerned, the results seem to indicate that the evolution is worse in those where the closure has not been performed.

Some variables have been identified as predictors of mortality in other studies with older patients, but not in ours: increased age [5, 7, 9, 12, 13], operative urgency [7], no cancer excision vs resection [7], living in an institution [10], and male sex [9]. Regarding age, although some authors have reported poorer outcomes in older patients [9, 24], others have not found differences in prognosis as a function of age [4, 25], so further studies are needed to provide more evidence on the evolution of cancer in this patient profile [26, 27].

Studies on colorectal cancer prognosis regardless of age limit have not found very different prognostic variables to those already mentioned for older patients [28–31]. Specifically, the predictors found in these studies were the following: tumour site (mixed results), advanced tumour stage, blood transfusion, older age, high grade, male sex, Chinese ethnicity, high carcinoembryonic antigen levels, emergency surgery, bowel obstruction, blood or lymphatic vessel invasion, and positive radial margins.

The variables mentioned so far are mainly clinical and related to the evolution of patients after the intervention. However, in our mortality prediction model at 5 years, other variables have been identified that evaluate the follow-up of these patients, in terms of the use of health resources such as specialist consultations, tests, treatments, emergency visits, or readmissions. In addition to the variables mentioned above, our study has identified others that predict mortality between 2 and 5 years from diagnosis, such as colostomy closure and readmissions during the years following surgery. There are already guidelines and recommendations on medium–long-term follow-up of these patients [32, 33], some of which indicate that survival increases when more intensive follow-ups are performed [33]. Therefore, it is important that future studies consider this type of variable so that health services can make decisions about the surveillance to be carried out on these patients.

Another difference between our study and others is that we have found identifying predictors of mortality in colorectal cancer is the follow-up period. Most other studies have chosen shorter follow-up periods, and only Hessman et al. [10] presented data for 5 years after the surgery. That is, our study provides mortality prediction information on a relatively long follow-up period.

This study presents some strengths that should be highlighted. First of all, it is a large prospective cohort study, with 18 participating hospitals, which ensures more variability. Notably, the main studies we have mentioned in comparisons with our results were all retrospective. Second, this cohort has been followed for 5 years, which is a considerably long period that allows observing the evolution of these patients and detecting other factors that influence mortality, in addition to those more directly related to the surgery. Third, we have identified common variables on which data can be obtained easily as predictors of mortality, and these have been combined into single scores, providing information on patient prognosis, which could help in the decision-making process regarding treatment and/or follow-up. Finally, most previous studies focused on the prediction of mortality from colorectal cancer in older patients have used multivariable models, but have not explored other ways of analysing, interpreting, and presenting the data. In our study, we have developed a risk score, which could help clinicians interpret the data more easily. Only Heriot et al. [7] conducted this kind of analysis, and they followed patients until just 30 days after surgery, not considering longer-term factors.

On the other hand, we should also recognise some limitations of our research. First, as in any prospective study, missing data is a source of bias. We attempted to reduce this bias by training the reviewers in each centre, to strengthen consistency in the collection of the information. Secondly, we have presented the results together for colon and rectal cancer, despite some authors having pointed out special features of these types of cancer that make them suitable for separate analysis [34, 35]. Most of the studies we have found in the literature present results for colorectal cancer, and as do some of the reviews and reports that study these types of cancer in older patients [4]. Besides, we have not found significant differences in our results when considering cancer localisation. Future studies should investigate this issue, with larger sample sizes and multicentre approaches, seeking to obtain more generalizable data. Finally, an external validation would help confirming the robustness of the risk models that have been developed.

## Conclusions

The risk scores developed could be easily employed by clinicians as prediction rules helping the decision-making process following at long term older colorectal cancer patients as they contain important information that could condition decisions that need to be made at that point regarding, for example, which treatments to administer and when to follow these patients in order to reduce mortality.

## Data Availability

The datasets used and/or analysed during the current study are available from the corresponding author on reasonable request.
